# A 20(S)-protopanoxadiol derivative overcomes multi-drug resistance by antagonizing ATP-binding cassette subfamily B member 1 transporter function

**DOI:** 10.18632/oncotarget.7011

**Published:** 2016-01-25

**Authors:** Gang Chen, Junhua Liu, Wantao Chen, Qin Xu, Meng Xiao, Lihong Hu, Li Mao, Xu Wang

**Affiliations:** ^1^ Department of Oral and Maxillofacial-Head and Neck Oncology, Ninth People's Hospital, Shanghai Jiao Tong University, School of Medicine, Shanghai 200011, P. R. China; ^2^ Shanghai Key Laboratory of Stomatology and Shanghai Research Institute of Stomatology, Shanghai 200011, P. R. China; ^3^ Shanghai Research Center for Modernization of Traditional Chinese Medicine, Shanghai Institute of Material Medica, Chinese Academy of Sciences, Shanghai 201203, P. R. China; ^4^ Department of Oncology and Diagnostic Sciences, University of Maryland School of Dentistry, Baltimore, MD 21201, USA

**Keywords:** multi-drug resistance, ABCB1, chemotherapy, apoptosis, panax ginseng

## Abstract

In cancer cells, failure of chemotherapy is often caused by the ATP-binding cassette subfamily B member 1 (ABCB1), and few drugs have been successfully developed to overcome ABCB1-mediated multi-drug resistance (MDR). To suppress ABCB1 activity, we previously designed and synthesized a new series of derivatives based on 20(S)-protopanoxadiol (PPD). In the present study, we investigated the role of PPD derivatives in the function of ABC transporters. Non-toxic concentrations of the PPD derivative PPD12 sensitized ABCB1-overexpressing cells to their anti-cancer substrates better than either the parental PPD or inactive PPD11. PPD12 increased intracellular accumulation of adriamycin and rhodamine123 in resistant cancer cells. Although PPD12 did not suppress the expression of ABCB1 mRNA or protein, it stimulated the activity of ABCB1 ATPase. Because PPD12 is a competitive inhibitor, it was predicted to bind to the large hydrophobic cavity of homology-modeled human ABCB1. PPD12 also enhanced the efficacy of adriamycin against ABCB1-overexpressing KB/VCR xenografts in nude mice. In conclusion, PPD12 enhances the efficacy of substrate drugs in ABCB1-overexpressing cancer cells. These findings suggest that a combination therapy consisting of PPD12 with conventional chemotherapeutic agents may be an effective treatment for ABCB1-mediated MDR cancer patients.

## INTRODUCTION

Multi-drug resistance (MDR), which is the resistance to anti-cancer chemotherapeutic agents with different targets, reduces the efficacy of cancer chemotherapy [1, 2]. There are a variety mechanisms underlying MDR in cancer, including the induction of anti-apoptotic machinery, increased drug efflux, and reduced drug uptake. Among these causes, over-expression of adenine triphosphate (ATP)-binding cassette (ABC) transporters is the most common reason for MDR in various types of tumors [3–5].

Although there are several different ABC transporters associated with the development of MDR, ABCB1, ABCG2 and ABCC1 are the most important [6–10]. ABCB1 was the first human ABC transporter implicated in MDR [11–13]. ABCB1 expression is a marker of chemo-resistance and decreased survival in leukemia, lymphoma, osteosarcoma, small-cell lung cancer, ovarian cancer, breast cancer, and other malignancies [14]. Using the energy of ATP hydrolysis, ABCB1 pumps out a wide spectrum of chemotherapeutic drugs from tumor cells, including the taxanes, epipodophyllotoxins, vinca alkaloids and anthracyclines [15]. Therefore, suppressing the activity or decreasing the expression of ABCB1 could restore the sensitivity of MDR cancer cells to chemotherapeutic agents and allow for successful chemotherapy in patients with MDR due to ABCB1-overexpression [16]. Several chemicals can sensitize MDR cells to chemotherapeutic drugs, with promising preclinical and early clinical data [17, 18]. Unfortunately, several critical problems, including cytotoxic effects, altered drug pharmacokinetics, and adversely modified drug distribution have prevented these chemicals from a successful translation to the clinic.

Ginsenosides are the active ingredients of *Panax ginseng*, which has been used as a dietary supplement in Asia for over two thousand years. Within the ginsenoside family, ginsenoside Rg3 has been reported to reverse ABCB1-mediated MDR [19, 20]. Ginsenoside Rg3 is metabolized to 20(S)-protopanoxadiol (PPD) by intestinal bacteria, and this metabolite also has been reported to have anti-cancer activity and via inhibition of ABCB1. Given the extremely low toxicity of the compound, PPD is an attractive candidate as a chemo-sensitizer for treatment of MDR tumors [21–24]. To improve the effects of PPD, we previously designed and synthesized a series of PPD derivatives with aromatic substituted aliphatic amine at the 24 positions [25]. In this study, we demonstrate that PPD12, compared to its parental compound PPD and inactive compound PPD11, sensitizes ABCB1-medidated MDR cancer cells to chemotherapeutic agents *in vitro* and *in vivo*.

## RESULTS

### PPD12 enhances the sensitivity of MDR cells to ABCB1-substrates

**Figure 1:**
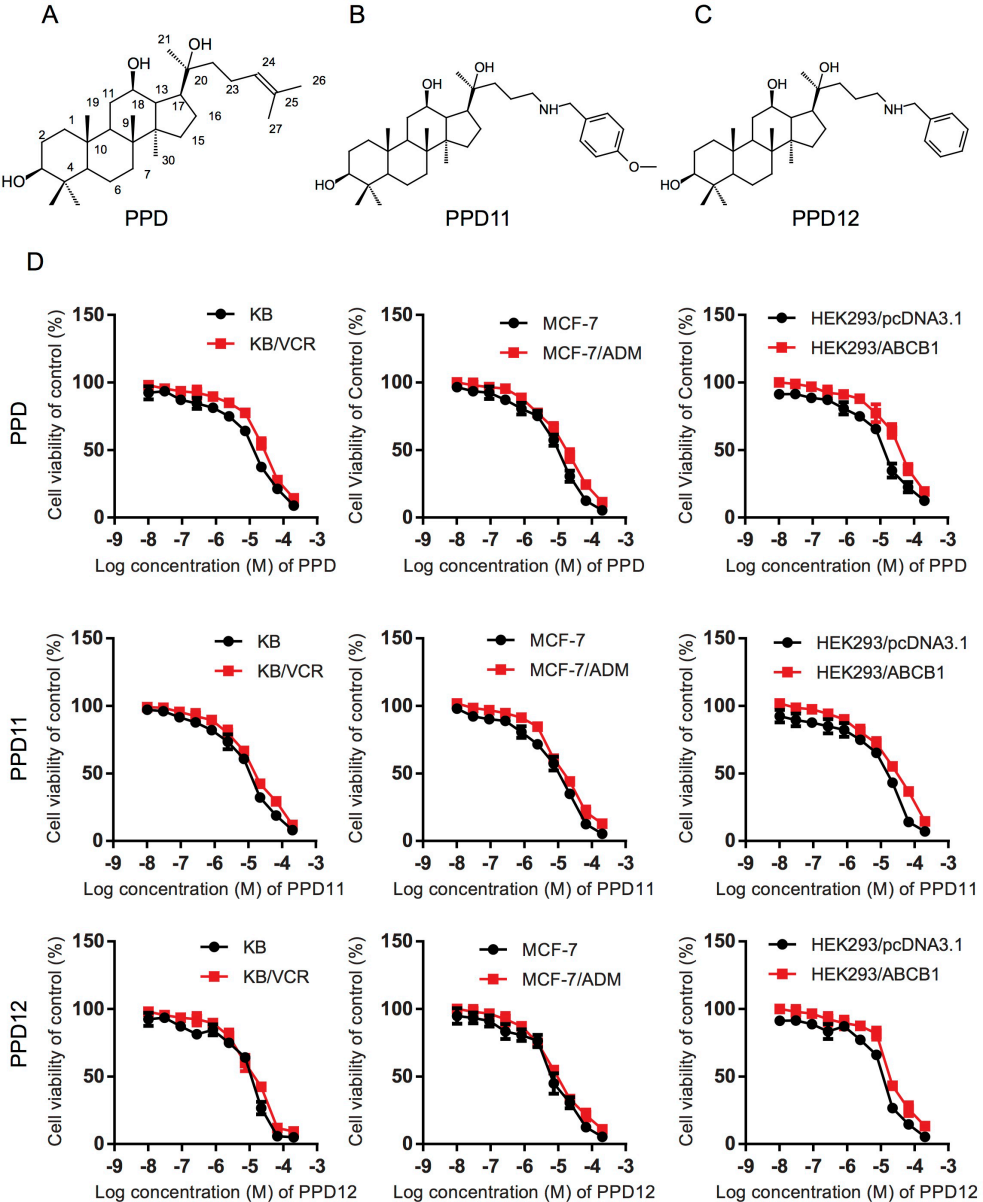
Influence of PPD, PPD11 and PPD12 on the cell viability of chemotherapy-sensitive and multi-drug resistant (MDR) cancer cells, respectively (**A**–**C**) The chemical structure of PPD, PPD11, and PPD12. (**D**) Cytotoxicity of PPD derivatives in pairs of KB and KB/VCR cells, MCF-7 and MCF-7/ADM cancer cells, HEK293 cells and HEK293 cells with ectopically-expressed ABCB1.

To reproduce MDR, we performed an MTT assay and confirmed that ABCB1-overexpressing cell lines (KB/VCR in Table 1, MCF-7/ADM in Table 2 and HEK293/ABCB1 in Table 3) have a higher tolerance for chemotherapeutic agents than their parental lines, making them suitable to evaluate the anti-MDR efficacy of PPD derivatives. To investigate the anti-proliferative effects of PPD derivatives (chemical structures shown in Figure 1A–1C) on MDR cancer cells, we first examined the cytotoxicity of PPD, PPD11, and PPD12 in three ABCB1-overexpressing cell lines with the MTT assay. Although there were differences in susceptibility to PPD, PPD11, and PPD12 (Figure 1D), all the IC_50_ values were higher than 6 μM. Therefore, a concentration of 5 μM was selected as the maximum working concentration for these derivatives in subsequent assays. We next tested the cytotoxicity of the PPD derivatives in combination with the ABCB1 substrate Adriamycin (ADM) and the non-ABCB1 substrate Cisplatin (CDDP) in KB, MCF-7 and HEK293 cells. The summary IC_50_ values are shown in Tables 1–3. Compared with PPD and PPD11, PPD12 exhibited a reversal effect to ADM but not to CDDP. PPD12 dose-dependently overcame resistance to ADM in both KB/VCR and MCF-7/ADM cells, but not in KB or MCF-7 cells. The known ABCB1 inhibitor Verapamil had similar effects in reverse. Furthermore, PPD12 did not alter the cytotoxicity of CDDP in any of the cells. Thus, thee one-carbon linear chain in the aromatic substituted aliphatic amine in the PPD12 was more effective than PPD in reversing MDR (Figure 1C), while PPD11, the analog with 4-methoxy- benzenamine substitution, was unable to overcome MDR (Tables 1–3).

**Table 1:** Effect of PPD derivatives on reversing ABCB1-mediated drug resistance of KB cells

Compounds	IC_50_ ± SD (μM)^a^ (fold reversal^b^)			
	KB		KB/VCR(ABCB1)	
**Adriamycin**	0.972 ± 0.273	(1.00)	6.245 ± 1.192	(1.00)
+ 1.25 μM PPD12	0.917 ± 0.054	(1.06)	1.469 ± 0.032	(4.25)*
+ 2.5 μM PPD12	0.876 ± 0.063	(1.11)	1.174 ± 0.025	(5.32)*
+ 5 μM PPD12	0.667 ± 0.075	(1.46)	0.483 ± 0.196	(12.92)**
+ 10 μM Verapamil	0.565 ± 0.016	(1.72)	0.474 ± 0.164	(13.17)**
**Paclitaxel**	0.073 ± 0.004	(1.00)	5.174 ± 0.421	(1.00)
+ 1.25 μM PPD12	0.054 ± 0.004	(1.33)	1.487 ± 0.097	(3.48)*
+ 2.5 μM PPD12	0.051 ± 0.006	(1.42)	1.080 ± 0.083	(4.79)*
+ 5 μM PPD12	0.050 ± 0.003	(1.36)	0.729 ± 0.011	(7.10)*
+ 10 μM Verapamil	0.038 ± 0.005	(1.92)	0.319 ± 0.029	(16.22)**
**Cisplatin**	2.755 ± 0.314	(1.00)	3.637 ± 0.325	(1.00)
+ 1.25 μM PPD12	1.583 ± 0.054	(1.74)	2.186 ± 0.504	(1.66)
+ 2.5 μM PPD12	1.524 ± 0.264	(1.81)	2.043 ± 0.273	(1.78)
+ 5 μM PPD12	1.458 ± 0.297	(1.89)	2.865 ± 0.956	(1.27)
+ 10 μM Verapamil	1.951 ± 0.504	(1.41)	3.075 ± 4.884	(1.18)
**Adriamycin**	0.557 ± 0.061	(1.00)	6.775 ± 0.511	(1.00)
+ 1.25 μM PPD11	0.583 ± 0.054	(0.96)	2.547 ± 0.504	(2.66)*
+ 2.5 μM PPD11	0.524 ± 0.064	(1.06)	2.437 ± 0.273	(2.78)*
+ 5 μM PPD11	0.458 ± 0.097	(1.22)	2.072 ± 0.956	(3.27)*
+ 10 μM Verapamil	0.451 ± 0.054	(1.24)	0.446 ± 0.084	(15.18)**
**Paclitaxel**	0.044 ± 0.003	(1.00)	3.217 ± 0.526	(1.00)
+ 1.25 μM PPD11	0.048 ± 0.002	(0.91)	1.828 ± 0.443	(1.76)
+ 2.5 μM PPD11	0.042 ± 0.004	(1.04)	1.496 ± 0.572	(2.15)*
+ 5 μM PPD11	0.047 ± 0.006	(0.93)	1.041 ± 0.378	(3.09)*
+ 10 μM Verapamil	0.035 ± 0.005	(1.27)	0.218 ± 0.095	(14.73)**
**Cisplatin**	2.237 ± 0.923	(1.00)	5.596 ± 1.403	(1.00)
+ 1.25 μM PPD11	2.306 ± 0.721	(0.97)	4.441 ± 0.923	(1.26)
+ 2.5 μM PPD11	1.896 ± 0.567	(1.18)	4.085 ± 1.104	(1.37)
+ 5 μM PPD11	2.513 ± 0.778	(0.89)	4.909 ± 1.034	(1.14)
+ 10 μM Verapamil	1.849 ± 0.729	(1.21)	4.477 ± 0.921	(1.25)
**Adriamycin**	0.717 ± 0.055	(1.00)	5.345 ± 1.074	(1.00)
+ 1.25 μM PPD	0.739 ± 0.014	(0.97)	4.143 ± 0.946	(1.29)
+ 2.5 μM PPD	0.763 ± 0.075	(0.94)	2.582 ± 0.547	(2.07)*
+ 5 μM PPD	0.710 ± 0.034	(1.01)	2.419 ± 0.826	(2.21)*
+ 10 μM Verapamil	0.771 ± 0.054	(0.93)	0.386 ± 0.076	(13.83)**
**Paclitaxel**	0.057 ± 0.004	(1.00)	4.118 ± 0.725	(1.00)
+ 1.25 μMPPD	0.049 ± 0.002	(1.17)	3.778 ± 0.847	(1.09)
+ 2.5 μM PPD	0.054 ± 0.004	(1.06)	1.898 ± 0.252	(2.17)*
+ 5 μM PPD	0.063 ± 0.003	(0.91)	1.481 ± 0.637	(2.78)*
+ 10 μM Verapamil	0.041 ± 0.002	(1.39)	0.246 ± 0.048	(16.71)**
**Cisplatin**	2.384 ± 0.217	(1.00)	3.631 ± 0.458	(1.00)
+ 1.25 μMPPD	2.563 ± 0.154	(0.93)	3.425 ± 0.924	(1.06)
+ 2.5 μM PPD	2.458 ± 0.436	(0.97)	3.077 ± 0.617	(1.18)
+ 5 μM PPD	2.110 ± 0.927	(1.13)	3.782 ± 0.762	(0.96)
+ 10 μM Verapamil	2.148 ± 0.411	(1.11)	2.859 ± 0.719	(1.27)

a IC_50_ values are represented as mean ± SD of three independent experiments performed in triplicate.

b The fold-reversal of MDR (values given in parenthesis) was calculated by dividing the IC_50_ for cells with the anticancer drugs in the absence of inhibitor by that obtained in the presence of inhibitor. Cell survival was performed by MTT assay as described in “Materials and Methods”. Verapamil, MK571 and FTC were used as a positive control of ABCB1, ABCC1 and ABCG2 inhibitor, respectively.

* *p* < 0.05, significantly different from those obtained in the absence of inhibitor.

** *p* < 0.01, significantly different from those obtained in the absence of inhibitor.

**Table 2:** Effect of PPD derivatives on reversing ABCB1-mediated drug resistance of MCF-7 cells

Compounds	IC_50_ ± SD (μM)^a^ (fold reversal^b^)			
	MCF-7		MCF-7/ADM (ABCB1)	
**Adriamycin**	0.326 ± 0.082	(1.00)	5.032 ± 0.535	(1.00)
+ 1.25 μM PPD12	0.291 ± 0.043	(1.12)	1.184 ± 0.125	(4.25)*
+ 2.5 μM PPD12	0.245 ± 0.066	(1.33)	0.605 ± 0.047	(8.32)*
+ 5 μM PPD12	0.236 ± 0.056	(1.38)	0.316 ± 0.036	(15.92)**
+ 10 μM Verapamil	0.213 ± 0.026	(1.53)	0.311 ± 0.054	(16.17)**
**Cisplatin**	7.322 ± 0.426	(1.00)	10.561 ± 0.215	(1.00)
+ 1.25 μM PPD12	6.452 ± 1.233	(1.13)	11.263 ± 1.234	(0.94)
+ 2.5 μM PPD12	7.013 ± 0.953	(1.04)	10.275 ± 0.892	(1.03)
+ 5 μM PPD12	8.223 ± 0.832	(0.89)	9.216 ± 0.761	(1.15)
+ 10 μM Verapamil	7.260 ± 0.561	(1.00)	9.865 ± 1.953	(1.07)
**Adriamycin**	0.541 ± 0.029	(1.00)	7.329 ± 0.592	(1.00)
+ 1.25 μM PPD11	0.487 ± 0.017	(1.11)	3.817 ± 0.151	(1.92)
+ 2.5 μM PPD11	0.525 ± 0.016	(1.03)	3.079 ± 0.127	(2.38)*
+ 5 μM PPD11	0.501 ± 0.025	(1.08)	2.554 ± 0.093	(2.87)*
+ 10 μM Verapamil	0.582 ± 0.028	(0.93)	0.516 ± 0.022	(14.21)**
**Cisplatin**	11.241 ± 1.625	(1.00)	14.853 ± 2.771	(1.00)
+ 1.25 μM PPD11	8.851 ± 1.602	(1.27)	13.029 ± 1.534	(1.14)
+ 2.5 μM PPD11	9.948 ± 1.053	(1.13)	14.420 ± 2.192	(1.03)
+ 5 μM PPD11	7.206 ± 0.996	(1.56)	13.262 ± 1.562	(1.12)
+ 10 μM Verapamil	9.214 ± 0.981	(1.22)	14.282 ± 2.265	(1.04)
**Adriamycin**	0.517 ± 0.017	(1.00)	5.951 ± 0.859	(1.00)
+ 1.25 μM PPD	0.528 ± 0.022	(0.98)	3.021 ± 0.989	(1.97)
+ 2.5 μM PPD	0.512 ± 0.054	(1.01)	2.576 ± 0.643	(2.31)*
+ 5 μM PPD	0.502 ± 0.048	(1.03)	2.705 ± 0.711	(2.20)*
+ 10 μM Verapamil	0.483 ± 0.065	(1.07)	0.468 ± 0.081	(12.71)**
**Cisplatin**	5.721 ± 1.435	(1.00)	6.724 ± 1.235	(1.00)
+ 1.25 μMPPD	4.367 ± 0.981	(1.31)	5.797 ± 0.956	(1.16)
+ 2.5 μM PPD	5.108 ± 0.875	(1.12)	4.908 ± 0.947	(1.37)
+ 5 μM PPD	4.689 ± 1.057	(1.22)	5.253 ± 1.026	(1.28)
+ 10 μM Verapamil	4.614 ± 1.026	(1.24)	6.058 ± 1.254	(1.11)

a IC_50_ values are represented as mean ± SD of three independent experiments performed in triplicate.

b The fold-reversal of MDR (values given in parenthesis) was calculated by dividing the IC_50_ for cells with the anticancer drugs in the absence of inhibitor by that obtained in the presence of inhibitor. Cell survival was performed by MTT assay as described in “Materials and Methods”. Verapamil, MK571 and FTC were used as a positive control of ABCB1, ABCC1 and ABCG2 inhibitor, respectively.

* *p* < 0.05, significantly different from those obtained in the absence of inhibitor.

** *p* < 0.01, significantly different from those obtained in the absence of inhibitor.

**Table 3:** Effect of PPD derivatives on reversing ABCB1-mediated drug resistance of HEK293 cells

Compounds	IC_50_ ± SD (μM)^a^ (fold reversal^b^)			
	HEK293/pcDNA3.1		HEK293/ABCB1	
**Adriamycin**	0.376 ± 0.034	(1.00)	3.554 ± 0.251	(1.00)
+ 1.25 μM PPD12	0.410 ± 0.096	(0.92)	1.679 ± 0.495	(2.12)*
+ 2.5 μM PPD12	0.342 ± 0.034	(1.10)	0.668 ± 0.202	(5.32)*
+ 5 μM PPD12	0.406 ± 0.113	(0.93)	0.223 ± 0.192	(15.94)**
+ 10 μM Verapamil	0.395 ± 0.074	(0.95)	0.206 ± 0.092	(17.23)**
**Cisplatin**	3.255 ± 0.214	(1.00)	3.637 ± 0.325	(1.00)
+ 1.25 μM PPD12	3.128 ± 0.454	(1.04)	3.431 ± 0.504	(1.06)
+ 2.5 μM PPD12	3.577 ± 0.664	(0.91)	2.843 ± 0.273	(1.28)
+ 5 μM PPD12	1.871 ± 0.297	(1.74)	2.723 ± 0.956	(1.34)
+ 10 μM Verapamil	2.583 ± 0.437	(1.26)	3.247 ± 0.887	(1.12)
**Adriamycin**	0.477 ± 0.053	(1.00)	5.469 ± 1.022	(1.00)
+ 1.25 μM PPD11	0.446 ± 0.039	(1.07)	4.883 ± 1.095	(1.12)
+ 2.5 μM PPD11	0.430 ± 0.045	(1.11)	3.107 ± 0.812	(1.76)
+ 5 μM PPD11	0.438 ± 0.086	(1.09)	2.288 ± 0.793	(2.39)*
+ 10 μM Verapamil	0.376 ± 0.094	(1.27)	0.383 ± 0.047	(14.27)**
**Cisplatin**	5.934 ± 0.968	(1.00)	6.637 ± 1.054	(1.00)
+ 1.25 μM PPD11	5.029 ± 1.263	(1.18)	5.926 ± 0.907	(1.12)
+ 2.5 μM PPD11	5.160 ± 0.924	(1.15)	6.321 ± 1.053	(1.05)
+ 5 μM PPD11	4.710 ± 0.834	(1.26)	5.352 ± 0.934	(1.24)
+ 10 μM Verapamil	4.495 ± 1.023	(1.32)	5.226 ± 1.091	(1.27)
**Adriamycin**	0.528 ± 0.082	(1.00)	4.736 ± 0.751	(1.00)
+ 1.25 μM PPD	0.513 ± 0.077	(1.03)	4.267 ± 0.783	(1.11)
+ 2.5 μM PPD	0.463 ± 0.083	(1.14)	2.041 ± 0.525	(2.32)*
+ 5 μM PPD	0.480 ± 0.072	(1.10)	1.611 ± 0.192	(2.94)*
+ 10 μM Verapamil	0.503 ± 0.091	(1.05)	0.370 ± 0.087	(12.81)**
**Cisplatin**	5.026 ± 0.443	(1.00)	5.947 ± 0.734	(1.00)
+ 1.25 μM PPD	4.654 ± 0.402	(1.08)	5.774 ± 0.425	(1.03)
+ 2.5 μM PPD	4.787 ± 0.578	(1.05)	5.263 ± 0.573	(1.13)
+ 5 μM PPD	4.488 ± 0.527	(1.12)	4.997 ± 0.713	(1.19)
+ 10 μM Verapamil	4.697 ± 0.354	(1.07)	5.083 ± 0.512	(1.17)

a IC_50_ values are represented as mean ± SD of three independent experiments performed in triplicate.

b The fold-reversal of MDR (values given in parenthesis) was calculated by dividing the IC_50_ for cells with the anticancer drugs in the absence of inhibitor by that obtained in the presence of inhibitor. Cell survival was performed by MTT assay as described in “Materials and Methods”. Verapamil, MK571 and FTC were used as a positive control of ABCB1, ABCC1 and ABCG2 inhibitor, respectively.

* *p* < 0.05, significantly different from those obtained in the absence of inhibitor.

** *p* < 0.01, significantly different from those obtained in the absence of inhibitor.

To examine the influence of PPD derivatives on other ABC transporters, we also examined anti-MDR effects in ABCC1-overexpressing (HL60/ADM) and ABCG2-overexpressing (S1-Mi-80) cell lines. Both cell lines were more resistant to chemotherapeutic agents than their parent lines (Table 4). However, PPD12 at 5 μM concentration only slightly reduced resistance to ADM (also the substrate of ABCC1) in ABCC1-overexpressing (HL60/ADM) cells and to Topotecan (also the substrate of ABCG2) in ABCG2-overexpressing (S1-MI-80) cells (Table 4). We therefore focused on examining the effects of PPD12 on ABCB1-mediated MDR, rather than ABCC1- or ABCG2-mediated MDR.

**Table 4:** Effect of PPD derivatives on reversing ABCC1-mediated drug resistance in HL60 cells and ABCG2-mediated drug resistance in S1 cells

Compounds	IC_50_ ± SD (μM)^a^ (fold reversal^b^)			
	HL60		HL60/ADR (ABCC1)	
**Adriamycin**	0.246 ± 0.012	(1.00)	2.177 ± 0.251	(1.00)
+ 1.25 μM PPD12	0.222 ± 0.037	(1.11)	1.877 ± 0.572	(1.16)
+ 2.5 μM PPD12	0.228 ± 0.077	(1.08)	1.387 ± 0.663	(1.57)
+ 5 μM PPD12	0.254 ± 0.046	(0.97)	1.022 ± 0.427	(2.13)*
+ 50 μM MK571	0.241 ± 0.053	(1.02)	0.223 ± 0.077	(9.77)**
	**S1**		**S1-Mi-80 (ABCG2)**	
**Topotecan**	0.574 ± 0.094	(1.00)	6.458 ± 1.447	(1.00)
+ 1.25 μM PPD12	0.504 ± 0.113	(1.14)	5.125 ± 0.912	(1.26)
+ 2.5 μM PPD12	0.474 ± 0.072	(1.21)	2.713 ± 0.428	(2.38)*
+ 5 μM PPD12	0.449 ± 0.061	(1.15)	2.131 ± 0.827	(3.03)*
+ 2.5 μM FTC	0.542 ± 0.069	(1.06)	0.338 ± 0.054	(19.12)**

a IC_50_ values are represented as mean ± SD of three independent experiments performed in triplicate.

b The fold-reversal of MDR (values given in parenthesis) was calculated by dividing the IC_50_ for cells with the anticancer drugs in the absence of inhibitor by that obtained in the presence of inhibitor. Cell survival was performed by MTT assay as described in “Materials and Methods”. Verapamil, MK571 and FTC were used as a positive control of ABCB1, ABCC1 and ABCG2 inhibitor, respectively.

* *p* < 0.05, significantly different from those obtained in the absence of inhibitor.

** *p* < 0.01, significantly different from those obtained in the absence of inhibitor.

### PPD12 inhibits ABCB1 transport but does not change ABCB1 expression

**Figure 2:**
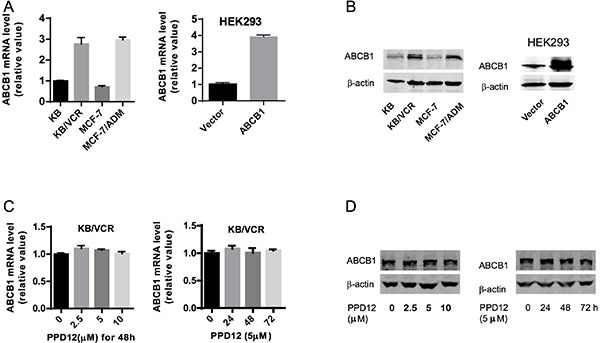
The effects of PPD12 on ABCB1 mRNA and protein levels in MDR cells (**A**) mRNA expression of *ABCB1* in KB, KB/VCR, MCF-7, MCF-7/ADM, HEK293, and HEK293-ABCB1 cells. (**B**) ABCB1 protein expression in KB, KB/VCR, MCF-7, MCF-7/ADM, HEK293, and HEK293-ABCB1 cells. (**C**) Neither varied concentrations of PPD12 after 48 h incubation nor 5 μM PPD12 for 72 h changed mRNA levels of *ABCB1* in multi-drug resistant KB/VCR cells. (**D**) Neither varied concentrations of PPD12 afer 48 h incubation nor 5 μM PPD12 for 72 h changed ABCB1 protein levels in multi-drug resistant KB/VCR cells.

Reversal of ABCB1-mediated MDR can be achieved either by inhibiting ABCB1 pump activity or by decreasing the expression of the protein [26]. To study the effect of PPD12 on ABCB1 expression, we measured both protein and mRNA levels using immunoblotting and real-time PCR analysis. KB/VCR and MCF-7/ADM cells had higher expression of ABCB1, while their parental cells had extremely low expression (Figure 2A–2B). Ectopic expression of ABCB1 was also higher in the HEK293/ABCB1 cells than the HEK293 cells (Figure 2A–2B). After a 48 h incubation with up to 10 μM PPD12 there was no effect on ABCB1 mRNA or protein levels in KB/VCR cells (Figure 2C–2D). We next performed a time-course experiment using 5 μM PPD12, and found no influence of this dose on ABCB1 levels even after a 72 h incubation (Figure 2C–2D).

**Figure 3:**
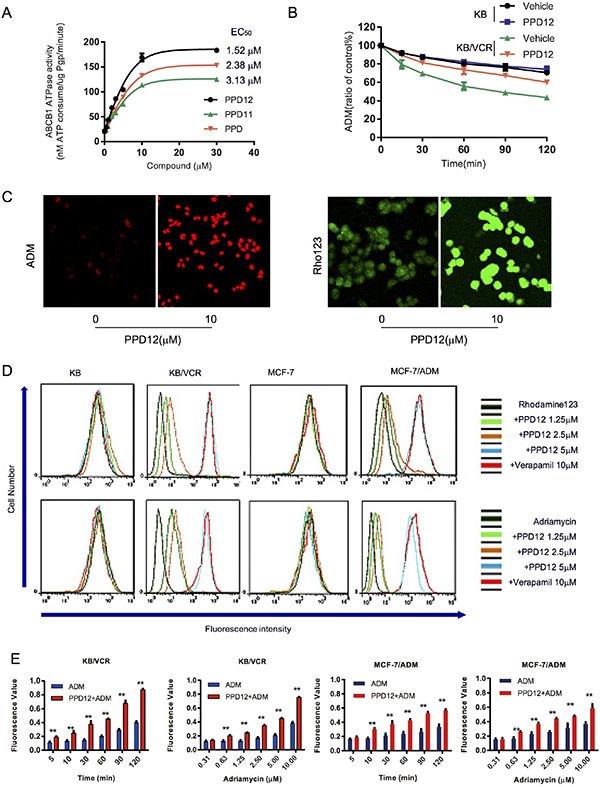
PPD12 inhibits the activity of ABCB1 in MDR cells (**A**) PPD12 enhances ABCB1 activity *in vitro* with EC_50_= 1.52 μM. (**B**) PPD12 decreases the efflux of ADM in KB/VCR cells, but not in KB cells. (**C**) PPD12 increases the concentration of ABCB1-substrates Adriamycin (ADM) and Rhodamine123 (Rho) in the KB/VCR cells. (**D**) PPD12 elevates the concentration of ABCB1-substrates in the MDR KB/VCR and MCF-7/ADM cells, but not their parental KB and MCF-7 cells. (**E**) 5 μM PPD12 suppresses the efflux of ADM in a time- and dose-dependent manner in KB/VCR and MCF-7/ADM cells.

We next examined the effects of PPD derivatives on the ATPase activity of ABCB1. The well-studied ABCB1 inhibitor, Verapamil, was used as a positive control, as this compound stimulates ABCB1-mediated ATP hydrolysis. As shown in Figure 3A, PPD12 enhanced the ATPase activity of ABCB1 in a dose-dependent manner with an EC_50_ value of 1.52 μM, while the original compound PPD stimulated ATPase activity with an EC_50_ value of 3.13 μM, suggesting that PPD12 is a substrate of ABCB1. Consistent with the data from the cell viability assay, the inactive derivative PPD11 induced ABCB1 activity with an EC_50_ value of 2.38 μM (Figure 3A). These data indicate that PPD12 acts as a competitive inhibitor to reduce the transport function of ABCB1, without altering mRNA or protein expression.

### PPD12 increases the accumulation of ADM and Rho123 in ABCB1-overexpressing cells by inhibiting the efflux of ABCB1 substrates

To investigate whether intracellular ADM accumulation is mainly through inhibition of efflux, we designed a time-course study to measure ADM efflux in the absence or presence of PPD12 in KB/VCR and KB cells. The KB and KB/VCR cells were treated with 5 μM ADM for 3 h at 37°C, followed by two washes with ice-cold PBS and subsequently maintained in ADM-free medium at 37°C in the absence or presence of 5 μM PPD12. ADM accumulation in KB/VCR cells was decreased to 79.43%, 69.53%, 55.8%, 48.77% and 43.47% in the absence of PPD12 at the indicated time points. In contrast, the percentages of remaining ADM were 89.37%, 81.3%, 73.53%, 67.3% and 60.1%, when KB/VCR cells were incubated with 5 μM PPD12 (Figure 3B). These results indicate that PPD12 inhibits the efflux functions of ABCB1.

To examine whether PPD12 increases the accumulation of ABCB1 substrates in MDR cancer cells, we measured intracellular levels of ADM and Rho123 in the presence or absence of PPD12. Representative confocal images confirmed that PPD12 increases the accumulation of ADM and Rho123 in KB/VCR and MCF-7/ADM cells (Figure 3C). As shown in Figure 3D, ADM and Rho123 fluorescence was higher in the parental cells than the MDR cells. However, PPD12 incubation increased the fluorescent signals of ADM and Rho123 in KB/VCR and MCF-7/ADM cells without affecting the parental cells. In both KB/VCR and MCF-7/ADM cells, the ADM content of cells was increased after ADM treatment. However, 5 μM PPD12 in combination with ADM enhanced the accumulation of ADM in a time- and dose-dependent manner (Figure 3E). These data suggest that PPD increases the ADM content in MDR cells.

### PPD12 in combination with ABCB1-substrate chemotherapeutic agents induces apoptosis in ABCB1-overexpressing cells

**Figure 4:**
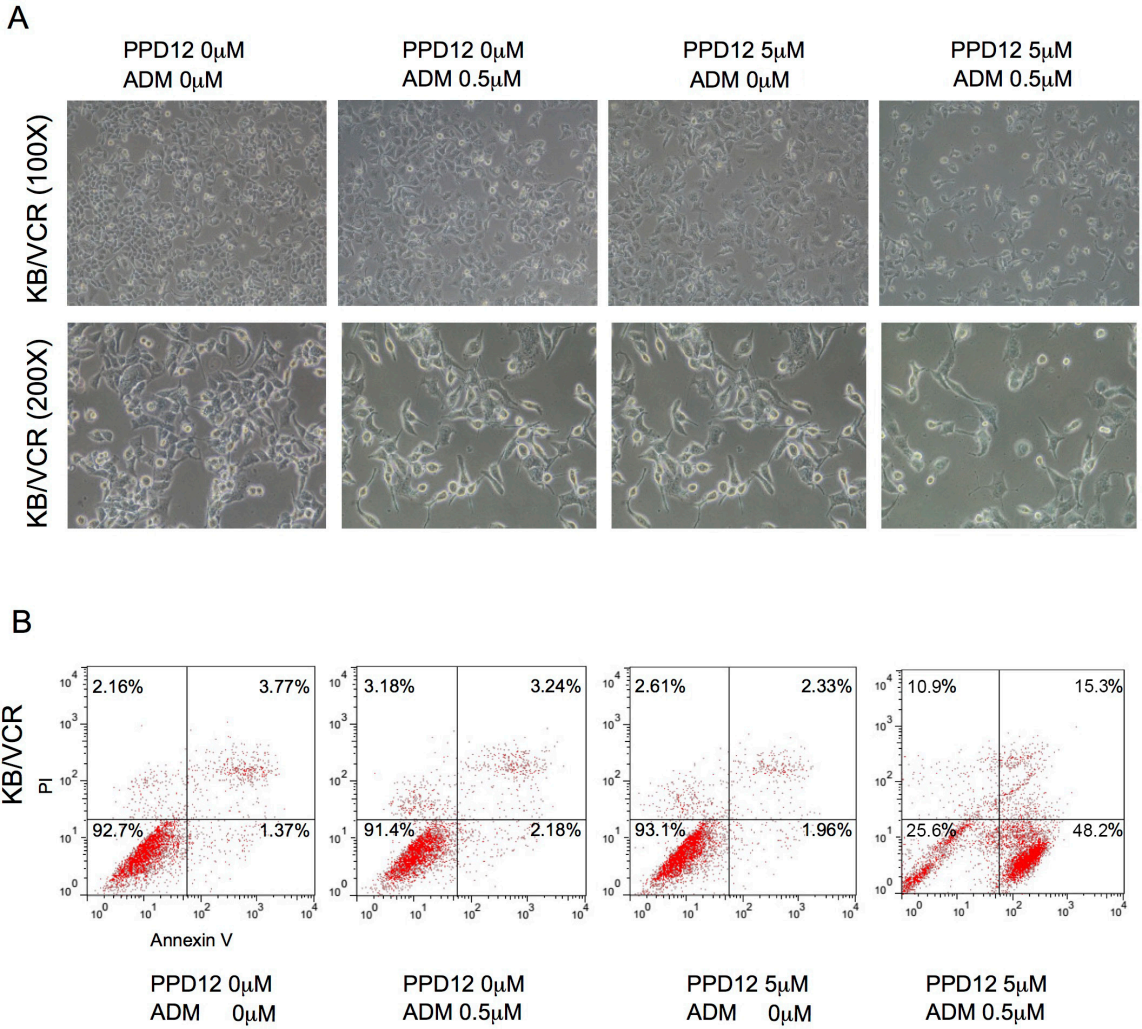
PPD12 enhances chemotherapeutic effects on ABCB1-expressing MDR cells (**A**) Representative microscopic images to show that 5 μM PPD12 incubation for 48 h acts synergistically with 0.5 μM ADM to inhibit cell viability of KB/VCR cells. (**B**) FACs analysis showing that 5 μM PPD12 treatment for 48 h enhances 0.5 μM ADM-induced apoptosis in the KB/VCR cells.

We next examined the effects of PPD12 in combination with chemotherapeutic agents, on cell apoptosis. Co-treatment with PPD12 and ADM enhanced apoptosis in comparison with PPD12 or ADM alone in KB/VCR cells but not in KB cells (Figure 4A). As shown in Figure 4B, co-treatment with PPD12 and ADM enhanced both early (Annexin V^+^/PI^−^) and late apoptosis (Annexin V^+^/PI^+^) in comparison with PPD12 or ADM alone in KB/VCR cells These data indicate that PPD12 enhances ADM-induced apoptosis.

### Model for binding of PPD12 to ABCB1

**Figure 5:**
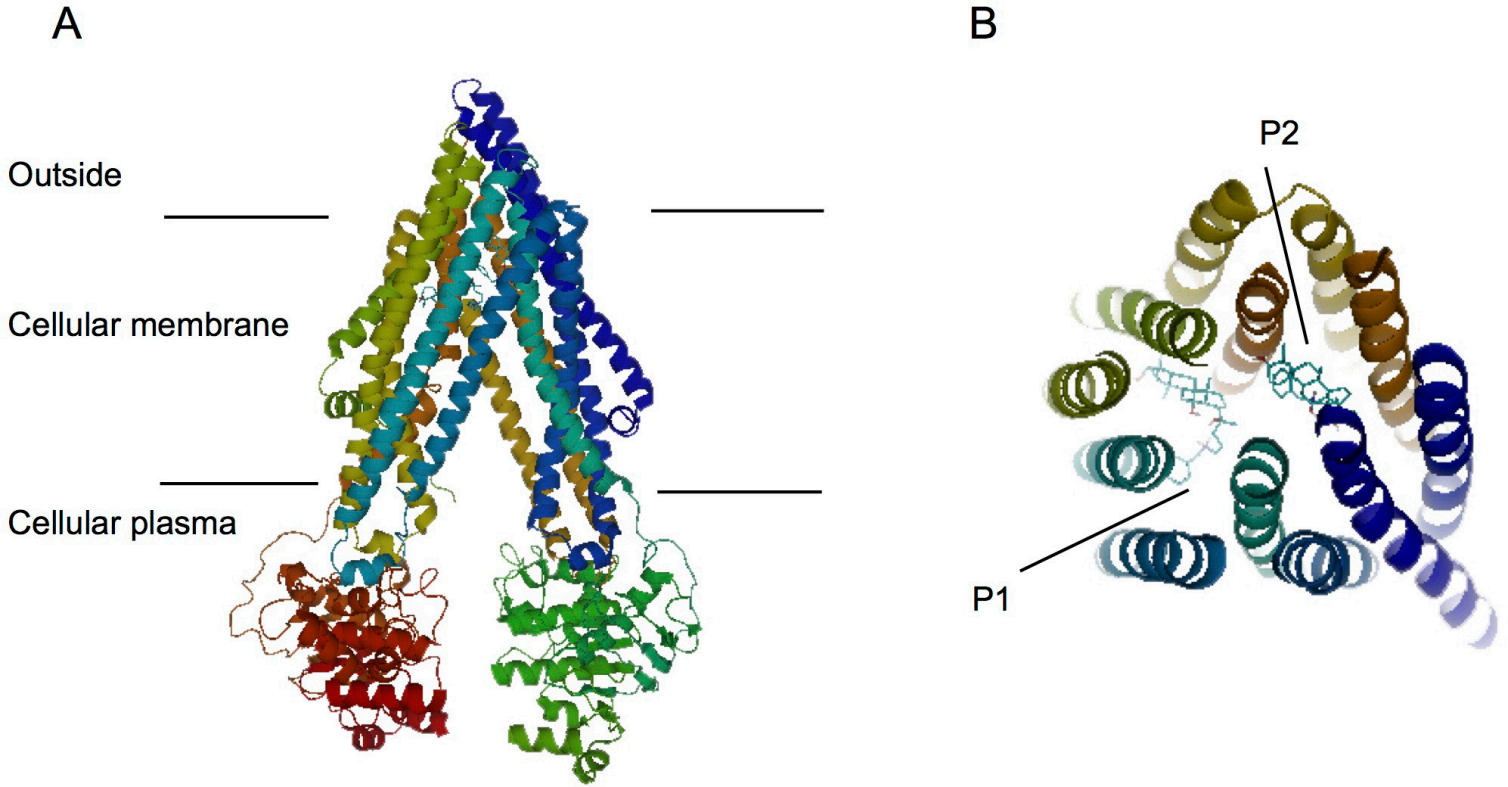
Computation of the docking of PPD12 to the ABCB1 crystal structure (**A**) Ribbon diagram of 3D structure conformation. (**B**) The optimal predicted binding mode of PPD12 within the mouse ABCB1 binding site is at two different binding sites (P1 and P2) with ABCB1 structure.

We next perforemd docking studies to understand the binding of PPD12 to ABCB1 at a molecular level, using the crystal structure of mouse ABCB1 as represented by ABCB1-PPD12 and ABCB1-Verapamil. As shown in Figure 5A–5B, the predicted binding mode showed hydrophobic interactions of PPD12 within the large drug-binding cavity of ABCB1. PPD12 was stabilized through hydrogen bonding and hydrophobic interactions with residues in the drug-binding pocket of ABCB1.

### PPD12 in combination with ADM inhibits the growth of KB/VCR xenografts in nude mice

**Figure 6:**
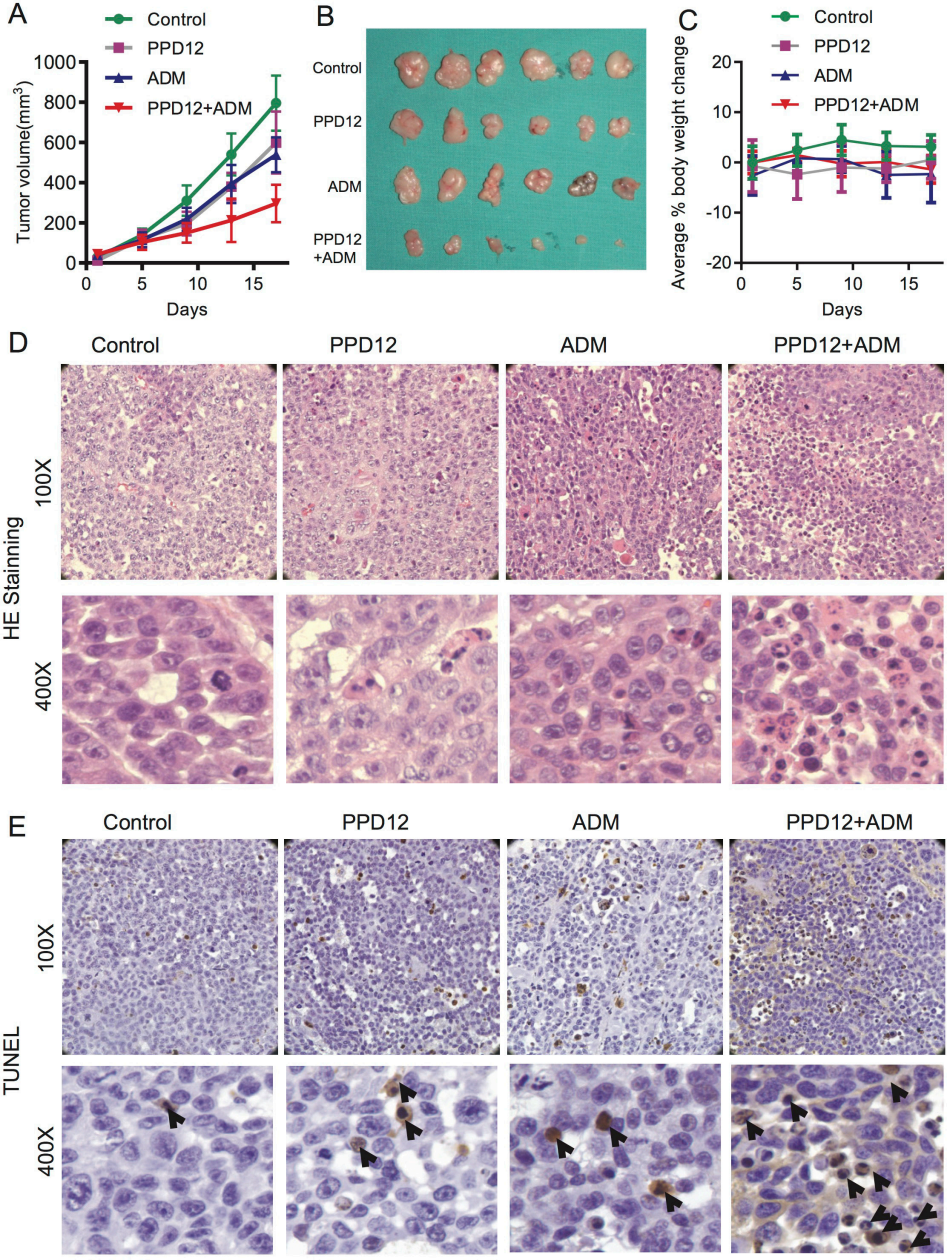
Co-treatment of PPD12 and chemotherapeutic drugs is more effective than single treatment in the KB/VCR xenograft model (**A**) Tumor volumes of each group of mice after 16 days. Mice received vehicle, oral treatment of 100 mg/kg PPD12, *i.p.* injection of 5 mg/kg ADM and oral treatment of 100 mg/kg PPD12 plus *i.p.* injection of 5 mg/kg ADM, respectively. (**B**) Representative images of xenografts in each group under different treatments. (**C**) Body weight changes of mice in each group. (**D**) HE staining demonstrates that PPD12 enhances ADM anti-cancer effects. (**E**) TUNEL staining indicates that co-treatment of PPD12 and Adriamycin induces apoptosis (indicated by black arrows) in the KB/VCR xenograft model.

To confirm the ability of PPD12 to antagonize ABCB1-mediated cancer MDR *in vivo*, we generated KB/VCR xenograft models in nude mice. As shown in Figure 6A–6B, treatment with PPD12 or ADM alone did not efficiently inhibit the growth of KB/VCR xenografts. PPD12 and ADM decreased the tumor growth at an inhibition ratio of 6.52 and 13.04, respectively (Table 5). In contrast, co-treatment with PPD12 and ADM inhibited the growth of KB/VCR xenografts with an inhibition ratio of 63.04% (Table 5). Moreover, there was no obvious loss of body weight in the combination group, suggesting that the combination regimen at the indicated dose did not cause toxicity in mice (Figure 6C). Additionally, H & E staining confirmed that the morphology changes of KB/VCR cells showed a round shape with decreased cell size and an increased nuclear-cytoplasmic ratio compared to control, ADM alone, or PPD12 alone groups (Figure 6D). TUNEL staining showed that the percentage of positive cells in KB/VCR xenografts was increased in the combination group in comparison with ADM alone or PPD12 alone (Figure 6E). These data indicate that PPD12 treatment overcomes MDR to ADM *in vivo*.

**Table 5:** Effect of PPD derivatives on reversing ABCB1-mediated drug resistance of KB/VCR cells *in vivo*

Group	Mean tumor volume (mm^3^)		Xenograft Mean Weigh (g)	IR (%)
	Start day 1	End day 17		
Control	27.9 ± 22.9	795.7 ± 455.1	0.46 ± 0.37	−
PPD12	11.8 ± 9.6	599.5 ± 435.8	0.43 ± 0.35	6.52
ADM	40.6 ± 36.4	538.6 ± 212.6	0.40 ± 0.34	13.04
Combination	43.5 ± 56.6	295.9 ± 245.7*	0.17 ± 0.18*	63.04*

* *P* < 0.05 significantly different from control group.

## DISCUSSION

Tumor cell MDR is a complicated and multi-faceted phenomenon and is the main cause of treatment failure in cancer chemotherapy [27]. MDR may originate from several biochemical mechanisms, but the primary cause is the over-expression of active drug efflux transporters, such as ABCB1, ABCG2 and ABCC1. These transporters pump anti-cancer drugs outside of cancer cells, reducing the efficacy of chemotherapeutic agents [28–30]. To reverse MDR, there have been numerous attempts to develop more effective drugs with acceptable tolerance [31]. Although many clinical trials have been conducted for specific targets, most results have been disappointing due to toxicity of the medicines themselves. The use of traditional herbal medicines has been gaining attention as an alternative method to overcome these shortcomings, as such medicines have many biological activities and low toxicity [32]. Drugs from natural plants have been used to cure cancer [33–36]. Among such compounds, PPD, an important metabolite of ginsenoside Rg3, is of particular interest [37, 38]. PPD has a high degree of anti-cancer activity, causing inhibition of ABCB1 in tumor cells and exhibiting extremely low toxicity [39, 40]. We improved the effectiveness of PPD by preparing a series of PPD analogues based on an analysis of the structure of ABCB1.

Because the crystal structure of human ABCB1 is currently unavailable, our docking simulations mainly relied on the homology model of newly refined mouse ABCB1. PPD12 is a hydrophobic molecule with a calculated LogP of 6.1. It also exhibits several aliphatic ring centers, which are critical for binding at the ABCB1 transporter. The hydrophobic nature of PPD12 may also contribute to its distribution within the cell membrane, while ABCB1 normally discharges substrates from the cellular membrane. Overall, docking simulation is helpful to provide clues to understand and optimize further PPD derivatives.

In conclusion, our results show that PPD12 antagonizes ABCB1-mediated cancer cell MDR *in vitro* and *in vivo* by directly blocking the drug-efflux functions of ABCB1. Our findings advocate the combined use of PPD12 with ABCB1 substrate anticancer drugs in the clinic to enhance chemotherapeutic responses.

## MATERIALS AND METHODS

### Chemical preparation

PPD, PPD11 and PPD12 were synthesized by our lab as previously reported [25] and prepared as a 100 mM stock solution in DMSO for *in vitro* studies. Verapamil (VRP), Adriamycin (ADM), Rhodamine 123 (Rho123), cisplatin (CDDP) and other chemicals were purchased from Sigma Chemical Co (St. Louis, MO, USA).

### Cell lines and cell culture

The cell lines utilized were: the human oral carcinoma cell line KB and its vincristine-selected ABCB1-overexpressing cell line KB/VCR, human breast carcinoma cell line MCF-7 and the ADM-resistant, ABCB1-overexpressing cell line MCF-7/ADM, human leukemia cell lines HL60 and its doxorubicin-selected ABCC1-overexpressing derivative HL60/ADM, human colon carcinoma cell line S1 and its mitoxantrone (MX)-selected ABCG2-overexpressing cell line S1-Mi-80, human primary embryonic kidney cell line HEK293 and its pcDNA3.1-ABCB1 stable-transfected cell line HEK293/ABCB1 (cultured in medium with 2 mg/ml G418). Cells were maintained in Dulbecco's Minimum Essential Medium (Invitrogen, Carlsbad, CA, USA) supplemented with 10% fetal bovine serum, 100 units/ml penicillin and 100 μg/ml streptomycin and incubated in a humidified atmosphere with 5% CO_2_ at 37°C. Drug-resistant cell lines were periodically cultured in the respective drug to confirm their resistance.

### Cell viability assay

Cell viability was assessed with an MTT assay as previously described [41]. In brief, cells were seeded in 96-well micro culture plates for 12 h to allow for attachment and were then incubated for 72 hours with various concentrations of PPD derivatives in the presence or absence of chemotherapeutic agents. MTT was then added to each well, and the cells were incubated for 4 h. The colored formazan product was quantified photometrically at 490 nm in a multi-well plate reader (Bio-Rad Laboratories, Hercules, CA, USA). The degree of resistance was estimated by dividing the IC_50_ for the MDR cells by that of the parental sensitive cells. Then, 10 μM VRP (inhibitor for ABCB1), 50 μM MK571 (inhibitor for ABCC1) and 2.5 μM FTC (inhibitor for ABCG2) were used in place of PPD12 as positive controls to confirm the mechanism of drug resistance in the MDR cell line models.

### Confocal microscopy

Resistant cells were cultured (1 × 10^4^ cells/well) on sterilized glass cover slips on the day prior to the assay. Cells were incubated with either 10 μM ADM alone or 10 μM ADM in the presence of 5 μM PPD12 in DMEM media for 1 h at 37°C. To examine the Rho123 accumulation, cells were incubated with either 10 μM Rho123 alone or 10 μM Rho123 in the presence of 10 μM PPD12 in DMEM media for 1 h at 37°C. The cells were then fixed with 4% paraformaldehyde. Nuclear staining was achieved by incubating cells in Hoechst 33342 for 5 min. The cells were then examined under a Confocal microscope (TCS SP2, Leica, Germany). The data presented were from one representative experiment of at least 3 independent repeats.

### Adriamycin and Rho 123 accumulation assay

First, cells were treated with PPD12 at various concentrations at 37°C for 24 h. Then, 10 μM ADM or 10 μM Rho123 was added to the medium and 1-h incubation was continued, respectively. After that, the cells were collected, washed twice with ice-cold PBS, and analyzed with flow cytometric (FCM) analysis (Beckman Coulter, Cytomics FC500, USA). VRP was used as a positive control inhibitor of ABCB1.

### Measurement of the cellular accumulation of adriamycin

The accumulation of ADM was measured as described previously [27]. Briefly, KB/VCR and MCF-7/ADM cells were plated at 1 × 10^4^ cells/well in 96-well plates. The cells were incubated with ADM at designed concentrations or time in the presence or absence of PPD12 for 2 h in a CO_2_ incubator at 37°C. After incubation, the medium was removed by aspiration, and the cells were washed with ice-cold PBS and lysed with 1% sodium dodecyl sulphate in PBS. Fluorescence intensity was measured with a microplate fluorimeter at emission wavelengths of 485 nm and 590 nm, respectively. Protein concentrations were measured by the Lowry method using a Bio-Rad BCA protein assay kit with bovine serum albumin as the standard. Accumulation ratios were calculated using the accumulation of ADM in cells incubated without PPD12 as a control.

### ADM efflux studies

ADM efflux was determined following a modification of methods described previously [26]. Briefly, KB and KB/VCR cells were treated with 5 μM ADM for 3 h at 37°C. The cells were then washed twice with ice-cold PBS and subsequently maintained in ADM-free medium at 37°C, in the absence or presence of 5 μM PPD12. Thereafter, at 0, 15, 30, 60, 90 and 120 min, cells were collected and washed again twice with ice-cold PBS, and analyzed with flow cytometric analysis.

### Apoptosis assay

Cells were harvested and washed twice with PBS, stained with Annexin V-FITC and propidium iodide (PI) in the binding buffer, and detected by FCM after 15 min incubation at room temperature in the dark. Fluorescence was measured at an excitation wavelength of 480 nm through FL-1 (530 nm) and FL-2 filters (585 nm). Apoptotic cells were quantified with the FlowJO software.

### Western blot analysis

Cells were harvested in SDS lysis buffer (Beyotime, Haimen, China), and cell lysates were electrophoresed through 8%–10% polyacrylamide gels and transferred to a PVDF membrane. The membranes were incubated with the desired primary antibody for ABCB1 (1:300, Boster, Wuhan, Hubei, China) and β-actin (1:5000, Sigma-Aldrich) overnight at 4°C, followed by incubation with the appropriate secondary antibody for 1 h at room temperature. The immunoreactive bands were visualized by the Odyssey^®^ Infrared Imaging System (Bioscience, San Diego, CA, USA). Detection of β-actin was used as a loading control.

### Real-time quantitative PCR

Total RNA was extracted from cells using TRIzol reagent (Invitrogen, USA) according to the manufacturer's instructions. Complementary DNA corresponding to 1 μg of total RNA was used per reaction in a quantitative PCR reaction performed on an ABI 7300 (Applied Biosystems, Foster City, CA, USA) and using Power SYBER Green Master Mix (Takara, Tokyo, Japan) and the following primers: ABCB1 sense 5′-CCC ATC ATT GCA ATA GCA GG-3′, anti-sense 5′-TDT TCA AAC TTC TGC TCC TGA-3′; β-actin sense 5′-GAT GAG ATT GGC ATG GCT TT-3′, anti-sense 5′-GAG AAG TGG GGT GGC TT-3′. Data were analyzed using the 2^−ΔΔCT^ method and normalized by β-actin expression in each sample.

### ABCB1 ATPase activity assay

The activity of ABCB1 ATPase in response to PPD derivatives was determined by the Pgp-Glo assay system (PromegaV3591, Madison, WI, USA). Briefly, according to the protocol of the manufacturer, the membrane vesicles (1.25 mg/ml/well) were incubated in different concentrations of the compound at 37°C for approximately 5 min. The ATPase reaction was initiated by the addition of Mg-ATP for incubating at 37°C for 30 min. After that, 50 μl of ATP Detection Reagent was added to all wells for initiating luminescence that is read on a plate-reading luminometer (PerkinElmer, Boston, MA, USA). Verapamil serves here as a positive control for drug stimulation of P-gp ATPase activity. A set of Mg-ATP standards at different concentrations was included in the assay for plotting a luminescent ATP standard curve and converting RLUs to specific activity expressed in units of nmoles ATP consumed/μg Pgp/min.

### Experimental animals

Twenty-four male SPF BALB/c nude mice (nu/nu, aged 5–6 weeks, and weighing approximately 20 g) were purchased from Shanghai Laboratory Animal Center (Shanghai, China) and housed under pathogen-free conditions in the animal care facilities of Ninth People's Hospital, Shanghai Jiao Tong University School of Medicine. Animal welfare and experimental procedures were in compliance with the Guide for Care and Use of Laboratory Animals (The Ministry of Science and Technology of China, 2006) and the related ethical regulations of the hospital. The Laboratory Animal Care and Use Committees of the hospital approved all experimental procedures.

With the KB/VCR cells, the inoculated nude mouse tumor xenograft model was established. Briefly, KB/VCR cells (1 × 10^6^) were injected subcutaneously in the right flank in the nude mice. The mice were randomized into one of four groups (eight animals in each group) after the xenograft reached a mean diameter of 0.5 cm, and then they received various regimens: (a) Control group (0.9% saline); (b) PPD12 group (biw × 6, p.o., 100 mg/kg); (c) ADM group (biw × 6, i.p., 5 mg/kg) and (d) PPD12 group (biw × 6, p.o., 50 mg/kg) plus ADM (biw × 6, i.p., 5 mg/kg). If the xenograft reached a volume of 1000 mm^3^, the animal was anaesthetized. Tumor size and animal weight were monitored every 4 days. Tumor volume was calculated using the formula: (length × width^2^/2). The mice were anaesthetized at the end of the experiment, and tumor tissue was excised from the mice and weighed. The rate of inhibition (IR) was calculated according to the formula:

$$\text{IR}=\left(1-\frac{\begin{array}{c} \text{Mean tumor weight of experimental group} \end{array}}{\begin{array}{c} \text{Mean tumor weight of control group} \end{array}}\right)\times 100\%$$

### HE and TUNEL assay

Tumor tissues were fixed in 4% formaldehyde, dehydrated with gradient ethanol, and embedded in paraffin. The tissue sections (4 μm) were de-waxed and rehydrated according to a standard protocol. For histological analysis, the sections were stained with hematoxylin and eosin. For the TUNEL assay, an *in situ* apoptosis detection kit (Roche Diagnostics, Branchburg, NJ) was used to detect apoptotic cells. The positive cells were identified, counted (three random fields per slides), and analyzed through ECLIPSE 80i light microscopy (Nikon, Tokyo, Japan).

### Docking protocol

The 3D structure of PPD12 was obtained from the software ChemDraw 7.0. The refined crystal structure of mouse ABCB1 in complex with QZ59-RRR (PDB ID: 4M2S) and QZ59-SSS (PDB ID: 4M2T) was obtained from the RCSB Protein Data Bank. Docking experiments were performed with Discovery Studio 3.0. The top scoring pose-ABCB1-complex was then subjected to energy minimization and used for graphical analysis.

### Statistics

Microsoft Office Excel 2010 and Prism 5.0 software were used in data processing and analyses. Results are shown as means ± SD. All experiments were repeated at least three times and the differences were determined using Student's *t*-tests. Statistical significance was *p < 0.05, **p < 0.01.
